# Evaluation of preoperative speed of progression and its association with surgical outcomes in primary congenital glaucoma patients: a retrospective study

**DOI:** 10.1186/s12886-017-0565-5

**Published:** 2017-09-18

**Authors:** Chunyu Guo, Yue Wu, Li Xu, Mao Li, Zi Wang, Ni Ni, Wenyi Guo

**Affiliations:** Department of Ophthalmology, Ninth People’s Hospital, Shanghai JiaoTong University School of Medicine, No. 639, Zhizaoju Road, Shanghai, 200011 People’s Republic of China

**Keywords:** Primary congenital glaucoma, Trabeculotomy, Speed of progression index

## Abstract

**Background:**

Surgeries are inevitable for treating primary congenital glaucoma (PCG) and risk factors of surgical failure play a key role in surgical decision making. The aim of this study was to investigate the influence of delay of surgery and preoperative speed of progression (SP) on the surgical outcomes in these patients.

**Methods:**

Medical records of 83 eyes of 51 PCG patients with trabeculotomy within 3 years were retrospectively observed. Surgical outcomes, demographic and clinical data were compared after separating the eyes into two groups based on the interval (between onset of PCG and trabeculotomy) and SP index (SPI) respectively. Student’s t-test, Wilcoxon rank-sum test, Pearson’s chi-square test and Kaplan-Meier survival analysis were used in the statistical analysis.

**Results:**

Comparative analysis showed better outcomes in the group with longer interval and lower SPIs. Better intraocular pressure (IOP) control was found in patients with lower SPI at 1, 3, 6, 12 and 24 months postoperatively (19.54 ± 4.84 mmHg vs. 24.75 ± 8.87 mmHg, *p* = 0.004; 19.88 ± 7.78 mmHg vs. 23.19 ± 6.74 mmHg, *p* = 0.089; 17.45 ± 6.23 mmHg vs. 21.31 ± 7.28 mmHg, *p* = 0.031; 15.09 ± 6.21 mmHg vs. 19.18 ± 6.66 mmHg, *p* = 0.008; 14.95 ± 2.95 mmHg vs. 18.10 ± 3.96 mmHg, p = 0.004). The correlation between SPI and IOP at 1, 3, 6, 12 and 24 months postoperatively was 0.328 (CI = 0.105 to 0.529, *p* = 0.005), 0.192 (CI = −0.070 to 0.429, *p* = 0.149), 0.261 (CI = 0.010 to 0.481, *p* = 0.042), 0.046 (CI = −0.183 to 0.270, *p* = 0.70), and 0.230 (CI = −0.072 to 0.493, *p* = 0.134), respectively. Patients with lower SPI were less likely to fail (χ2 = 22.71, *p* = 0.000, OR: 0.174; 95%CI: 0.059–0.510). Kaplan-Meier analysis showed a much slower decline of success rate in patients with lower SPI (χ2 = 25.52, p = 0.000).

**Conclusions:**

In PCG patients, lower preoperative SPI was associated with better short-term IOP control and success rate. Evaluation of preoperative SPI may help with surgical decision. However, early detection and treatment are important given the same SPI.

## Background

Despite the low incidence, primary congenital glaucoma (PCG) is the most common glaucoma in newborns and infants. Approximately 18% of children in institutions for the blind have this disease and 5% of all pediatric blindness is attributed to it [1]. Anti-glaucoma medicines elicit low response from PCG patients before surgery, and they are mainly used as adjunctive therapy [2]. Therefore, surgical intervention becomes inevitable in the life of a PCG patient. Investigations into potential risk factors of surgical failure have been conducted, which can help with surgical decision making [3–9]. Although delay of surgery was associated with worse prognosis of PCG patients by many researchers, unfortunately, it is still commonly found in both developed and developing countries [3, 10], and the influence of such a delay on prognosis was scarcely investigated [1, 11–13]. Severity of PCG was reported to influence the surgical outcomes [4]. However, the severity of PCG evaluated in their study only represented the preoperative status of PCG but the speed of progression (SP) of the disease in preoperative period was not taken into consideration.

In this study, we retrospectively reviewed the medical charts of PCG patients who received trabeculotomy as their first surgical treatment, attempting to investigate the influence of delay of surgery and SP on the surgical outcomes.

## Methods

This retrospective study was approved by the Institutional Review Board of our institute, and the investigations were carried out in accordance with the Declaration of Helsinki.

### Patients

The medical records of all patients who were diagnosed with PCG, underwent trabeculotomy as a primary surgery and had regular visits with one of the authors before June 1, 2015 were retrospectively reviewed. The patients who met the glaucoma definition with isolated angle anomalies were enrolled in this study, as described in the ninth World Glaucoma Consensus [14]. The patients who underwent surgery while over 3 years of age, had a follow-up less than 1 year, or possessed history of any former intraocular surgery or other ocular anomalies were excluded.

### Surgery and data collection

All surgeries were performed by one surgeon. The trabeculotomy technique was based on the description by Harms and Dannheim [15]. Demographic data and results from basic ophthalmic examinations under anaesthesia or sedation (EUA/S) were retrieved. EUA/S included biomicroscopy, measurement of intraocular pressure (IOP), measurement of corneal diameter, and evaluation of cup-to-disc ratio (C/D ratio), and these examinations were performed both preoperatively and at each follow-up visit. However, preoperative C/D ratio could only be identified in less than 30% of eyes due to corneal opacity. IOP was measured with an ICARE apparatus (Icare Finland, Helsinki, Finland) or Tonopen (Reichert, Depew, NY, USA) under anaesthesia or sedation. The average of three consecutive readings was recorded as the result of one examination. Evaluation of the C/D ratio was made by a single doctor, ensuring a high level of consistency. The severity scores of PCG before surgery were calculated according to Al-Hazmi [4]. A score from 1 to 3 was given for parameters including IOP, corneal diameter, and corneal opacity. IOP under 25 mmHg and over 35 mmHg were given 1 and 3, respectively. Corneal diameter under 13 mm and over 14.5 mm were given 1 and 3, respectively. Good and poor corneal opacities were given 1 and 3, and fair was given 2. We used an index to represent the actual SP of PCG, which was calculated by dividing the total score by the age at surgery (months). The onset age was recorded as the exact age when the parents noticed the abnormalities or the age at initial presentation was used instead. The days between onset of PCG and trabeculotomy was converted to months and recorded as the interval. The reasons for the longer interval in patients with an interval over 1 month and first detected sign of PCG were obtained from parents when the patients presented in our clinic for the first time.

### Success and failure

Surgical success was defined as postoperative IOP ≤ 21 mmHg without medicine or secondary surgery and no evidence of disc cup or cornea enlargement at the final visit. A failure was recorded when the IOP was over 21 mmHg, a secondary surgery was needed, or an obvious enlargement of the disk cup or cornea was observed during the follow-up.

### Statistical analysis

Data were analysed with SPSS statistical software (Statistical Package for the Social Sciences, version 17.0, SPSS Inc., Chicago, IL, USA). Continuous variables were evaluated with Student’s t-test or Wilcoxon rank-sum test, while categorical variables were evaluated by Pearson’s chi-square test. Data were summarized using means and standard deviation or medians and interquartile ranges (IQR) for continuous variables and proportions for categorical variables. The cumulative success rate was evaluated by a Kaplan-Meier survival analysis and compared using the Log-Rank test. A probability value (*p*-value) < 0.05 was considered statistically significant.

## Results

A total of 83 eyes were included in the final analysis. The interval was within 1 month for 37 eyes and over 1 month for the other 46. The main reason for longer intervals was ignorance about PCG (11/46), followed by unclear diagnosis (8/46) and inability of treatment in the former hospital (7/46). The most common first sign of PCG noticed by parents was corneal opacity (28/83), followed by corneal enlargement (17/83) and photophobia (12/83).

### Influence of interval between onset of PCG and trabeculotomy on surgical outcomes

**Fig. 1:**
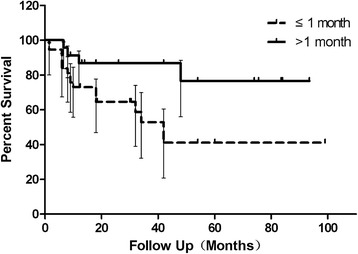
Kaplan-Meier survival curves for success in the groups with different intervals. The success rate of primary congenital glaucoma patients after trabeculotomy declined much more slowly in patients with intervals over 1 month. The interval is defined as the time between detection and surgery

Better IOP control was found in patients with longer intervals at 1, 3, and 6 months postoperatively (Table 1). Kaplan-Meier analysis showed a much slower decline of success rate in patients with longer intervals (Fig. 1). Log-Rank test revealed a statistically significant difference between two groups (χ2 = 8.754, *p* = 0.003). Success rate at the final visit was higher (82.6% vs. 54.1%) in the longer interval group (χ2 = 7.944, *p* = 0.005, odds ratio (OR): 4.04; 95% confidence interval (95% CI): 1.49–10.97). Better postoperative C/D ratio was observed in the group with longer intervals (0.6 (IQR 0.5–0.8) vs. 0.9 (IQR 0.8–0.9)) However, no significant difference was found in IOP between groups in the final visit (16.50 mmHg (IQR 15.00–20.25 mmHg) vs. 18 mmHg (IQR 13.00–31.00 mmHg), *p* = 0.696).

**Table 1:** Comparison of intraocular pressure between groups with different intervals

	Interval≦ 1 month (mmHg)	Interval > 1 month (mmHg)	*p*-Value
1 month postoperatively	24.74 ± 4.83(*n* = 31)	19.67 ± 8.13(*n* = 42)	0.003^a*^
3 months postoperatively	24.74 ± 6.95(*n* = 27)	19.06 ± 6.72(n = 31)	0.003^a*^
6 months postoperatively	22.16 ± 6.89(*n* = 25)	17.61 ± 6.57(*n* = 36)	0.012^a*^
12 months postoperatively	18.00(13.00–20.00)(n = 31)	16.00(12.65–17.3)(*n* = 44)	0. 340^b^
18 months postoperatively	16.00(13.75–19.00)(*n* = 26)	15.00(14.00–19.5)(*n* = 29)	0.879^b^
24 months postoperatively	16.00(13.50–19.00)(*n* = 21)	16.00(14.00–18.00)(*n* = 23)	0.795^b^

^a^Student’s t-test; interval: interval between onset to surgery; ^b^Wilcoxon rank sum; *: *p* < 0.05

### Comparison of demographic and clinical data between groups with different intervals

Disparities in terms of demographic data and well-reported risk factors between groups may interfere with the outcomes, but no significant difference was observed with regard to initial IOP, corneal diameter, follow-up period, existence of Haab’s striae, severity of corneal opacity, gender, laterality or severity score (Table 2). The median age at surgery was 6.5 months (IQR 2.5–11.125 months) in patients with longer intervals, compared to 2.5 months (IQR 1.5–4 months) in patients with shorter intervals (Z = −4.35; *p* = 0.00), and the age at surgery was strongly correlated with interval length (*r* = 0.779, CI = 0.677–0.852, *p* = 0.000). Age at onset was even smaller in patients with longer intervals than those with shorter intervals (1 month (IQR 0–2 months) vs. 2 month (IQR 1–3 month); Z = −3.403; *p* = 0.001). However, the SP index (SPI) in the group with longer intervals was smaller than that observed for the other group (Table 2).

**Table 2:** Comparison of demographic data and risk factors of surgical failure between groups

	Interval ≤ 1 month (*n* = 37)	Interval > 1 month (*n* = 46)	p-Value
Initial IOP (mmHg)	34.87 ± 5.29	32.24 ± 9.21	0.108^a^
Corneal diameter (mm)	13.18 ± 1.33	13.38 ± 0.78	0.412^a^
Follow-up period (month)	30 (15.25–42)	34 (16.75–75.5)	0.324^b^
Gender (female:male)			0.440^c^
Female	13 (35.1%)	20 (39.8%)	
Male	24 (64.9%)	26(60.2%)	
Laterality			0.993^c^
Bilateral	33 (89.2%)	41 (89.1%)	
Unilateral	4 (10.8%)	5 (10.9%)	
Haab’s striae			0.418^c^
Yes	21 (56.8%)	22 (47.8%)	
No	16 (43.2%)	24(52.2%)	
Severity of corneal opacity			0.066^c^
Good	2 (5.4%)	11 (23.9%)	
Fair	30 (81.1%)	29 (63.0%)	
Poor	5 (13.5%)	6 (13.0%)	
Severity score			0.397^c^
Mild	0 (0.00%)	0 (0.00%)	
Moderate	20 (54.1%)	32 (69.6%)	
Severe	17(45.9%)	14(30.4%)	
SPI	2.80(1.78–3.33)	0.93(0.48–2.33)	0.000^b^

^a^Student’s t-test; ^b^Wilcoxon rank sum test; ^c^Chi-square test; *interval*: interval between onset to surgery; *IOP* intraocular pressure, *SPI* speed of progression index

### Influence of preoperative speed of progression of PCG on surgical outcomes

**Fig. 2:**
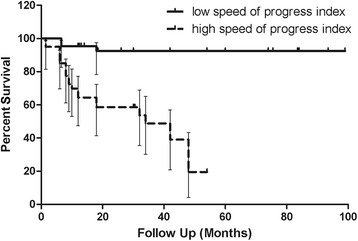
Kaplan-Meier survival curves for success in the groups with high and low preoperative speed of progression index. The success rate of primary congenital glaucoma patients after trabeculotomy declined much more slowly in the group with lower speed of progression index

We separated all patients into two groups based on SPI. Better IOP control was found in patients with lower SPI at 1, 3, 6, 12 and 24 months postoperatively (19.54 ± 4.84 mmHg vs. 24.75 ± 8.87 mmHg, *p* = 0.004; 19.88 ± 7.78 mmHg vs. 23.19 ± 6.74 mmHg, *p* = 0.089; 17.45 ± 6.23 mmHg vs. 21.31 ± 7.28 mmHg, *p* = 0.031; 15.09 ± 6.21 mmHg vs. 19.18 ± 6.66 mmHg, *p* = 0.008; 14.95 ± 2.95 mmHg vs. 18.10 ± 3.96 mmHg, p = 0.004). The correlation between SPI and IOP at 1, 3, 6, 12, and 24 months postoperatively was 0.328 (CI = 0.105 to 0.529, *p* = 0.005), 0.192 (CI = −0.070 to 0.429, *p* = 0.149), 0.261 (CI = 0.010 to 0.481, *p* = 0.042), 0.046 (CI = −0.183 to 0.270, *p* = 0.70), and 0.230 (CI = −0.072 to 0.493, *p* = 0.134), respectively. Patients with lower SPI were less likely to fail (χ2 = 22.71, *p* = 0.000, OR: 0.174; 95%CI: 0.059–0.510). Kaplan-Meier analysis showed a much slower decline in success rate in patients with lower SPI (Fig. 2). Log-Rank test revealed a statistically significant difference between the two groups (χ2 = 25.52, p = 0.000). No significant difference was observed with regard to initial IOP, corneal diameter, existence of Haab’s striae, laterality or severity score between groups. The follow-up period was longer in patients with a lower SPI (42.00 months (IQR: 18.00–83.50 months) vs. 28.00 months (IQR: 12.00–42.00 months), *p* = 0.005).

## Discussion

Unlike adult glaucoma, childhood glaucoma is more challenging to ophthamologists with regard to distorted ocular anatomy, aggressive healing response, and higher anaesthesia risk. Trabeculotomy is designed to rebuild the connection between the anterior chamber and Schlemm’s canal, and IOP could be maintained under 14 mmHg for 2 years, with a success rate of over 87% in adults [16]. Although such a low IOP and a high success rate could not be achieved in PCG patients, as observed in our study, sufficiently controlled IOP, minimal complications, and a relatively short surgical duration make trabeculotomy the first-choice therapy in PCG.

Primary congenital glaucoma is caused by the malformation of the anterior chamber angle, and elevated IOP leads to irreversible glaucomatous retinal fibre impairment. Therefore, theoretically, the earlier the surgery is performed, the earlier the IOP is controlled and the less the retinal fibre will be damaged. However, in the present study, trabeculotomies performed over 1 month after the onset of PCG were associated with better IOP control, better cumulative success rates and smaller postoperative C/D ratios when compared to trabeculotomies performed within 1 month after onset. This was the opposite of the suggestion by Ikeda that delayed detection of onset or surgery might lead to progression of glaucoma [12]. Also, no obvious differences existing between groups with longer and shorter intervals could serve as an explanation in terms of demographic parameters and well-reported potential risk factors of surgical failure, including initial IOP [5, 6], corneal diameter [6], follow-up period [7–9], existence of Haab’s striae, severity of corneal opacity [3, 6], gender, laterality, age at onset or severity [3, 4, 6, 7]. In the attempt to find a reasonable explanation, it occurred to us that the evaluation of severity was based on preoperative examinations, and a similar severity with a shorter interval indicated more rapid progression of disease. The classification system of severity was proposed by Al-Hazmi and colleagues in a retrospective study including 820 eyes, and they found that severity was associated with surgical success. This system has been accepted and adopted by many researchers and paves the way for a quantitative evaluation of severity, making our evaluation of SPI possible [17–19]. The severity score could only represent the status of the disease right before surgery, while SPI could reflect how rapidly the disease had been progressing prior to the surgery. Former studies only focused on the progression after presentation or surgery in order to evaluate the surgical outcome instead of regarding SP as a predictive factor of prognosis. In a study reported by Yalvac [20], the efficacy of trabeculotomy in PCG patients operated on within 3 months was investigated. They found that the preoperative axial length was negatively associated with success rate. The duration between age of onset and surgery ranged from 10 to 30 days, less than 1 month. If we examined the speed that the axial length grows to substitute SPI, the success rate should be lower in those with higher SPI. In this way, their results support our results that the higher the SPI, the worse the outcomes. When evaluating the prognosis of PCG patients, both the severity and the SP should be taken into consideration, among other risk factors.

Patients with lower SPI had better prognosis than those with higher SPI, and earlier diagnosis and treatment would still benefit an individual patient. Anti-glaucoma medicines are used as adjunctive therapies in postoperatively uncontrolled cases, while surgery is regarded the first choice for initial treatment [2]. In 1967, clinical research suggested that early and accurate diagnosis would promise a better prognosis [21]. However, nearly half a century later, the average interval between onset of PCG and first surgery was still reported to range from 2 to 5 months [7, 22, 23]. As reported by Ben-zion, the average age at surgery was 3.3 years in Ethiopia. In our study, an interval of over 1 month was found in 46 out of 83 eyes, and a lower SPI was observed in this group. Patients with lower SPI tended to present later because parents had difficulties in detecting less severe anomalies. In this study, for all 11 eyes in which ignorance of PCG was the reason for a longer interval, the first detected sign was photophobia instead of more severe signs that could be easily detected, like corneal opacity or enlargement. However, for the 15 eyes with an unclear diagnosis or inability of treatment as the reasons for longer intervals, the first detected signs were corneal opacity or enlargement, suggesting that an inadequate awareness of PCG was present not only in parents but also in doctors. Our study highlighted again the importance of increasing awareness and educational programs about PCG for both the public and doctors, which could result in earlier detection and treatment for PCG patients.

There are three main limitations to our study. First, the strongest limitation is the retrospective design. We reviewed and discussed all medical charts to minimize bias. However, a further prospective study is needed to confirm the causative relationship between SPI and surgical outcomes. Second, the sample number was relatively small, which was a result of our narrow inclusion criteria. We excluded patients with trabeculotomy performed after 3 years of age because, by the age of two, the elasticity of the eye will reduce and scleral rigidity prevents a reliable comparison of severity involving the diameter of cornea [24]. Larger samples are needed to determine the influence of SPI on surgical outcomes in PCG. Second, the final visual acuity was not evaluated in this study. Zagora et al. reported that good IOP control does not necessarily predict good visual outcome in PCG [7]. In the present study, we mainly focused on the IOP control and success rate after trabeculotomy in patients with different intervals and SPIs. Additionally, PCG patients in our study were approximately 2 years old and had difficulties with the visual acuity test. We will continue to study these issues.

## Conclusions

In PCG patients, lower preoperative SPI was associated with better short-term IOP control and success rate. This result implies that preoperative SP assessment may help with surgery decision making. However, early detection and treatment are important if the preoperative SPIs are the same.

## Abbreviations

C/D ratio: Cup-to-disc ratio
EUA/S: Examinations under anaesthesia or sedation
IOP: Intraocular pressure
IQR: Interquartile ranges
PCG: Primary congenital glaucoma
SP: Speed of progression

## References

[1] Moore DB, Tomkins O, Ben-Zion I (2013). A review of primary congenital glaucoma in the developing world. Surv Ophthalmol.

[2] Quaranta L, Biagioli E, Galli F, Poli D, Rulli E, Riva I (2016). Latanoprost and Dorzolamide for the Treatment of Pediatric Glaucoma: The Glaucoma Italian Pediatric Study (Gipsy), Design and Baseline Characteristics. Adv Ther.

[3] Ben-Zion I, Tomkins O, Moore DB, Helveston EM (2011). Surgical results in the management of advanced primary congenital glaucoma in a rural pediatric population. Ophthalmology.

[4] Al-Hazmi A, Awad A, Zwaan J, Al-Mesfer SA, Al-Jadaan I, Al-Mohammed A (2005). Correlation between surgical success rate and severity of congenital glaucoma. Br J Ophthalmol.

[5] Levy J, Carmi R, Rosen S, Lifshitz T (2005). Primary congenital glaucoma presenting within the first three months of life in a Bedouin population: prognostic factors. J Glaucoma.

[6] Dietlein TS, Jacobi PC, Krieglstein GK (1999). Prognosis of primary ab externo surgery for primary congenital glaucoma. Br J Ophthalmol.

[7] Zagora SL, Funnell CL, Martin FJ, Smith JE, Hing S, Billson FA (2015). Primary congenital glaucoma outcomes: lessons from 23 years of follow-up. Am J Ophthalmol.

[8] Essuman VA, Braimah IZ, Ndanu TA, Ntim-Amponsah CT (2010). Combined trabeculotomy and trabeculectomy: outcome for primary congenital glaucoma in a West African population. Eye (Lond).

[9] Saltzmann RM, Reinecke S, Lin X, Cavanagh HD, Whitson JT (2012). Long-term outcomes of a pseudo 360-degree trabeculotomy ab externo technique for congenital glaucoma at children's medical center. Clin Ophthalmol.

[10] Alsheikheh A, Klink J, Klink T, Steffen H, Grehn F (2007). Long-term results of surgery in childhood glaucoma. Graefes Arch Clin Exp Ophthalmol.

[11] Shaffer RN (1982). Prognosis of goniotomy in primary infantile glaucoma (trabeculodysgenesis). Trans Am Ophthalmol Soc.

[12] Ikeda H, Ishigooka H, Muto T, Tanihara H, Nagata M (2004). Long-term outcome of trabeculotomy for the treatment of developmental glaucoma. Arch Ophthalmol.

[13] Mandal AK, Chakrabarti D (2011). Update on congenital glaucoma. Indian J Ophthalmol.

[14] Beck A, Chang TCP, Freedman S, Weinreb R, Grajewski A, Papadopoulos M, Grigg J, Freedman S (2013). Definition, Classification, Differential Diagnosis. Childhood Glaucoma The 9th Consensus Report of the World Glaucoma Association.

[15] Harms H, Dannheim R (1970). Epicritical consideration of 300 cases of trabeculotomy ‘ab externo’. Trans Ophthalmol Soc U K.

[16] Quaranta L, Hitchings RA, Quaranta CA (1999). Ab-interno goniotrabeculotomy versus mitomycin C trabeculectomy for adult open-angle glaucoma: a 2-year randomized clinical trial. Ophthalmology.

[17] Huang J, Lin J, Wu Z, Xu H, Zuo C, Ge J (2015). Outcomes of Ahmed glaucoma valve implantation in advanced primary congenital glaucoma with previous surgical failure. Clin Ophthalmol.

[18] Badeeb OM, Micheal S, Koenekoop RK, den Hollander AI, Hedrawi MT (2014). CYP1B1 mutations in patients with primary congenital glaucoma from Saudi Arabia. BMC Med Genet.

[19] Uva MG, Avitabile T, Reibaldi M, Bucolo C, Drago F, Quaranta L (2013). Long-term efficacy of latanoprost in primary congenital glaucoma. Eye (Lond).

[20] Yalvac IS, Satana B, Suveren A, Eksioglu U, Duman S (2007). Success of trabeculotomy in patients with congenital glaucoma operated on within 3 months of birth. Eye (Lond).

[21] Richardson KT, Ferguson WJ, Shaffer RN (1967). Long-term functional results in infantile glaucoma. Trans Am Acad Ophthalmol Otolaryngol.

[22] Eldaly MA (2014). Pneumatic trabecular bypass versus trabeculotomy in the management of primary congenital glaucoma. Graefes Arch Clin Exp Ophthalmol.

[23] Chen XL, Chen YH, Jin XH, Sun XH, Meng FR, Guo WY (2009). Trabeculotomy on primary congenital glaucoma: a retrospective study of 164 cases (257 eyes). Zhonghua Yi Xue Za Zhi.

[24] MacKinnon JR, Giubilato A, Elder JE, Craig JE, Mackey DA (2004). Primary infantile glaucoma in an Australian population. Clin Experiment Ophthalmol.

